# Batch Similarity Based Triplet Loss Assembled into Light-Weighted Convolutional Neural Networks for Medical Image Classification

**DOI:** 10.3390/s21030764

**Published:** 2021-01-24

**Authors:** Zhiwen Huang, Quan Zhou, Xingxing Zhu, Xuming Zhang

**Affiliations:** Key Laboratory of Molecular Biophysics of Ministry of Education, Department of Biomedical Engineering, School of Life Science and Technology, Huazhong University of Science and Technology, No 1037, Luoyu Road, Wuhan 430074, China; D201780516@hust.edu.cn (Z.H.); zhoudachuan@hust.edu.cn (Q.Z.); D201677473@hust.edu.cn (X.Z.)

**Keywords:** medical image classification, convolutional neural networks, batch similarity based triplet loss

## Abstract

In many medical image classification tasks, there is insufficient image data for deep convolutional neural networks (CNNs) to overcome the over-fitting problem. The light-weighted CNNs are easy to train but they usually have relatively poor classification performance. To improve the classification ability of light-weighted CNN models, we have proposed a novel batch similarity-based triplet loss to guide the CNNs to learn the weights. The proposed loss utilizes the similarity among multiple samples in the input batches to evaluate the distribution of training data. Reducing the proposed loss can increase the similarity among images of the same category and reduce the similarity among images of different categories. Besides this, it can be easily assembled into regular CNNs. To appreciate the performance of the proposed loss, some experiments have been done on chest X-ray images and skin rash images to compare it with several losses based on such popular light-weighted CNN models as EfficientNet, MobileNet, ShuffleNet and PeleeNet. The results demonstrate the applicability and effectiveness of our method in terms of classification accuracy, sensitivity and specificity.

## 1. Introduction

Medical image classification is one of the more basic and important tasks for computer-aided diagnosis (CAD). An efficient medical image classifier can help reduce the workload for the doctors and guide the inexperienced physicians. In recent years, deep learning (DL), especially the convolutional neural networks (CNNs)-based methods, have shown outstanding performance for image processing tasks. Therefore, some researchers have developed and applied lots of heavy-weighted CNNs for medical image classification [1]. For example, the AlexNet [2] is used for breast cancer recognition from histological images [3] and Alzheimer’s disease diagnosis from MRI images [4]. Besides this, the visual geometry group (VGG) network [5] is utilized to identity papillary thyroid carcinomas in cytological images [6] and discover COVID-19 cases based on X-ray images [7]. In addition, the Inception-V3 [8] is trained for distinguishing skin cancer images from normal ones [9], and differentiating benign and malignant renal tumors based on CT images [10]. Moreover, the residual network (ResNet) [11] is applied to HEp-2 cell classification [12] and the quality assessment of retinal OCT images [13]. Even though these mentioned heavy-weighted models can achieve better performance in some specific applications, they have limited capabilities in many medical applications in the case of small samples. The reason lies in the fact that the effectiveness of these networks depends on the quality and quantity of training data, while there are usually not enough annotated image data to train very deep networks. Therefore, such light-weighted networks as DenseNet [14], MobileNet [15], ShuffleNet [16] and EfficientNet [17] arouse researchers’ interest, and many models are applied to medical image classification tasks. For example, Yuan et al. used the DenseNet to realize polyp recognition from the wireless capsule endoscopy images [18]. Brehar et al. designed a new shallow CNN model to recognize hepatocellular carcinoma and it provided a higher accuracy than the compared deep models [19]. Besides this, some researchers tried to diagnose COVID-19 using MobileNet V2, ShuffleNet, and EfficientNet based on X-ray images [20,21,22]. These light-weighted models have less adjustable weights, are easier to train and have better computational efficiency compared with the heavy-weighted ones, but their classification ability still needs to be improved to deal with complex medical applications, especially in the case of small amounts of training samples. 

From the perspective of loss function, the mentioned CNN models take the traditional cross entropy (CE) as the loss function for the training process. However, the CE function only measures the difference between the predicted probability distribution and the target distribution [23], which means that CE-based models cannot analyze the distribution of samples and classes. Compared with regular CNN models, some models for few-shot recognition tasks adopt different losses where the deep metric learning (DML) technique is involved [24]. The DML losses take advantage of data distribution for discovering the differences among classes and finding the major common patterns for each category. In other words, the DML losses can assist the CE-based CNN models in the training process. 

Generally, the DML-based CNNs use multi-inputs and produce multiple embedding vectors so that they can calculate a certain distance metric from the embedding vectors as the loss for training. By minimizing the loss, the CNNs can generate similar embedding vectors for images belonging to the same class, and dissimilar vectors for different categories of inputs [25]. For example, the Siamese network [26] proposed by Jane et al. took paired images as inputs so that it can use the distance of embedding vectors along with the ground truth to form the contrastive loss function. Hoffer et al. [27] designed the triplet network which used the ternary input to obtain distinguishing features from two different kinds of samples. To get more information from different classes, Sohn et al. [28] proposed multi-class N-pair loss, which utilized one sample from each class to identify each input example. Notice that this pair-based DML loss only observes several samples one at a time, which means the estimated data distribution varies a lot. As an improvement, Song et al. proposed a lifted structured feature-embedding method [29], which measured all the distances between every two samples in a training batch so that it could learn to discriminate embedding vectors according to the data distribution of the input batch.

Despite the fact that the DML-based CNNs can analyze the data distribution and improve the training process, they are designed for such few-shot recognition or image retrieval tasks as face recognition [28,30]. Even though some researchers have tried to apply DML loss to train CNNs in such medical applications as coronary heart disease classification [31] and COVID-19 diagnosis [32], these models need extra classifiers to accomplish the classification tasks. Besides this, it is hard to select a reasonable support set for regular medical image classification tasks. There are two simple ways to introduce DML into regular classification applications without a query image set. One is to use DML loss to train the CNN models to obtain distinguishing embedding vectors, and classify them with a traditional classifier. For example, Gupta et al. [33] proposed Siamese CNN, which was trained based on the triplet loss, and used the SVM to recognize mitotic HEp-2 cell images. This kind of scheme relies on the classification ability of the adopted classifier in the case of a small training dataset. Another idea is to combine the DML loss with the CE loss to train models. However, these mentioned pair-based DML losses involve specially designed rules for pairing the samples. The rules are incompatible for regular CE-based CNNs at the training stage. Sun et al. [34] tried to combine triple loss with the CE loss to overcome the problem that the herbal images are too diverse and complicated. Lei et al. [35] proposed a novel class-center-involved triplet loss, and combined it with the CE loss to deal with the imbalanced data problem for the skin disease classification. However, these DML losses are still pair-based and cannot effectively represent the data distribution of the dataset.

**Figure 1:**
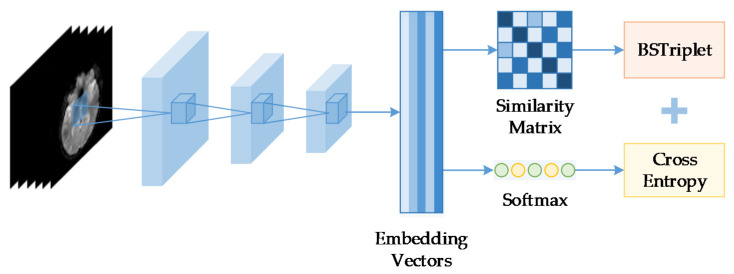
Framework of a convolutional neural network (CNN) trained by batch similarity-based triplet loss and cross entropy.

To more effectively utilize the DML loss to train the regular CNNs in the case of medical image classification applications, we have proposed a novel batch similarity-based triplet loss, denoted as “BSTriplet” loss for short. The proposed loss can facilitate assessing the similarities among a batch of input images instead of several pairs to evaluate the distribution of training data. As shown in Figure 1, the proposed BSTriplet loss takes the embedding vectors produced by the CNN model to calculate a similarity matrix, which contains all the similarities between every pair of two samples in the input batch. For each sample, the BSTriplet loss analyzes the similarities between it and the rest of the samples. According to the ground truth, the BSTriplet loss converts the difference of samples from the same class and the similarity of the rest of the samples into a loss. By reducing the produced loss, the CNN model can achieve the goal of making the samples of the same class become closer in the embedding space, or farther apart if they are from different categories. The BSTriplet loss calculates the loss for each sample in parallel, which means that it is compatible for model training by CE loss. Since the proposed loss can only play a role in clustering, we integrated the softmax for classification and utilized the CE to evaluate the ability of classification. Moreover, we have designed a novel sample-mining method to build the input batch according the distribution of the whole training dataset. In this way, the input batch will contain diverse samples of all classes, and still have better consistency compared with batches constructed by the random selection. To evaluate the effectiveness of the proposed loss, we have tried to use the BSTriplet loss combined with CE loss to train such popular light-weighted networks as MobileNet-V3 [36], ShuffleNet-V2 [37], EfficientNet [17] and PeleeNet [38] on three different medical image datasets. The results show that the BSTriplet loss can guide the CNN models to cluster the embedding vectors. Moreover, it can help these light-weighted CNN models to achieve better performance compared with the traditional CE loss or other combined losses. 

In this study, our main contributions are as follows:

1. We have introduced a novel batch similarity-based loss, which can be embedded into arbitrary CNN models to help the training process;
2. A reasonable sample mining strategy is designed to help CNN models in the better estimation of the distribution of the training dataset;
3. The proposed loss is combined with cross entropy to train several light-weighted CNN models, and its effectiveness has been demonstrated on different kinds of medical image datasets.

The context of this paper is organized as follows. Section 2 describes the details of the designed BSTriplet loss and the designed data mining technique. Section 3 presents the experiments performed to discover some characteristics of the proposed loss and compare it with some other DML loss. The conclusion is given in Section 4.

## 2. Method

### 2.1. Similarity-Based Triplet Loss

When training a CNN model F(·), a batch of samples X is given to the model in each iteration of the error back-propagation algorithm. For an arbitrary sample $x_{i}\in X$, one can obtain the embedding vector $f_{i}=F(x_{i})\in {\mathbb{R}}^{1\times l}$ of length l. Generally, the output of the pooling layer or flattening layer which follows the last convolutional layer is adopted as the embedding vector *f_i_*. We use $f_{i}^{+}$ to denote a vector corresponding to the sample $x_{i}^{+}$ belonging to the same class as $x_{i}$, and $f_{i}^{-}$ to denote the opposite case. As mentioned above, an ideal loss function can guide the embedding vector to get closer to those in the same class, and further away from the others. Therefore, we will consider the triplet loss in this research. The regular similarity-based triplet loss is defined as [39]

(1) $$\begin{array}{l} L_{trip}^{s}\left(s_{i}^{+},s_{i}^{-}\right)=\max\left(0,1-s_{i}^{+}+s_{i}^{-}\right)\\ i.\;e.\;s_{i}^{+}=\frac{f_{i}^{T}f_{i}^{+}}{{\left\|f_{i}\right\|}_{2}{\left\|f_{i}^{+}\right\|}_{2}},\;s_{i}^{-}=\frac{f_{i}^{T}f_{i}^{-}}{{\left\|f_{i}\right\|}_{2}{\left\|f_{i}^{-}\right\|}_{2}} \end{array}$$

where $s_{i}^{+}$ and $s_{i}^{-}$ are the similarity between $f_{i}$ and $f_{i}^{+}$, and that between $f_{i}$ and $f_{i}^{-}$, respectively. Accordingly, the embedding vectors $f_{i}$ are normalized via L2 normalization (i.e., ${\hat{f}}_{i}=f_{i}/{\left\|f_{i}\right\|}_{2}$) so that similarity $s_{i}^{+}$ can be expressed as $s_{i}^{+}={\hat{f}}_{i}^{T}{\hat{f}}_{i}^{+}$, where ${\hat{f}}_{i}^{T}$ means the transposition of ${\hat{f}}_{i}$. By reducing $L_{trip}^{s}$, $s_{i}^{+}$ and $s_{i}^{-}$ will be close to 1 and 0, respectively. Notice that for $f_{i}$, $L_{trip}^{s}$ only uses two reference vectors $f_{i}^{-}$ and $f_{i}^{+}$ to calculate the loss, which is disadvantageous for training due to the variety and complexity of the triplet inputs. 

### 2.2. Batch Similarity Based Triplet Loss

**Figure 2:**
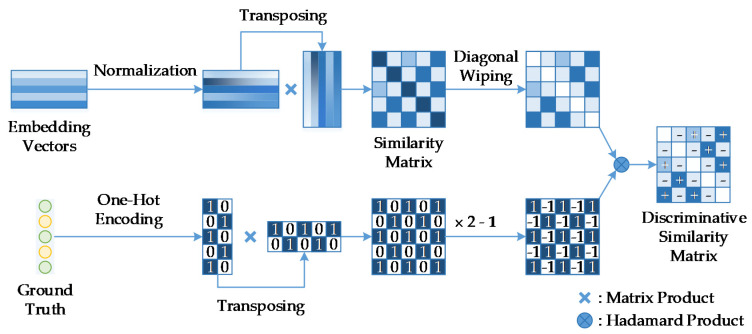
Diagram of calculation and analysis of the similarity matrix in the BSTriplet loss. The symbols “+” and “−” denote the similarities of positive pairs and negative pairs, respectively.

In the BSTriplet, we aim at making full use of the input batch. Therefore, similarities between every set of two corresponding samples in the batch are measured to evaluate the data distribution. After the embedding vectors $f\in {\mathbb{R}}^{N_{b}\times l}$ for the input batch containing $N_{b}$ samples are obtained, each vector $f_{i}$ is L2 normalized to produce ${\hat{f}}_{i}=f_{i}/{\left\|f_{i}\right\|}_{2}$, which is inherited from the traditional similarity-based triple loss. The similarity matrix $S\in {\mathbb{R}}^{N_{b}\times N_{b}}$ is produced by $S=\hat{f}{\hat{f}}^{T}$, which stores the similarities of all possible pairs of samples in the batch. To analyze the similarity matrix S, the ground truth $y\in {\mathbb{R}}^{N_{b}\times 1}$ is necessary. Each label $y_{i}\in {\mathbb{R}}^{1\times 1}$ can be transformed into a row vector ${\hat{y}}_{i}\in {\mathbb{R}}^{1\times C}$ according to the one-hot encoding method, where C is the number of classes. As a result, ${\hat{y}}_{i}$ is a row vector full of zeros, except the $y_{i}$-th element is one. A binary matrix $B\in {\mathbb{R}}^{N_{b}\times N_{b}}$ can be generated by $B=\hat{y}{\hat{y}}^{T}$. The binary matrix B is utilized to distinguish the similarities for positive pairs from those for negative ones by calculating the discriminative similarity matrix D:

(2) $$\begin{array}{c} D=S⊗B+S⊗(B-1)-S⊗I\\ =S⊗(2B-1)⊗I \end{array}$$

where the symbol ⊗ denotes the Hadamard product [40]. $1\in {\mathbb{R}}^{N_{b}\times N_{b}}$ and $I\in {\mathbb{R}}^{N_{b}\times N_{b}}$ are a matrix full of 1s and an identity matrix, respectively. Note that in the matrix D, the similarities of positive pairs are greater than 0, and vice versa. Moreover, the diagonal elements of D are zero. The diagonal elements of S will be wiped out in that they represent the similarity between the vectors $f_{i}$ and themselves, and they are helpless for evaluating data distribution due to the fact that $S_{i,i}=1$. The process of evaluating the similarities among all samples for the BSTriplet loss is shown in Figure 2. 

Based on the above discriminative similarity matrix D, the loss for each input sample $x_{i}\in X$ can be evaluated by observing the i-th row of the matrix D. The values $D_{i,j}$ in the i-th row represent the similarity between the vector $f_{i}$ and other vectors in the batch (except the diagonal element $D_{i,i}=0$). Clearly, there may be multiple similarities for positive and negative pairs in the i-th row. For convenience, we re-denote the similarities for positive pairs as $D_{i,j}^{+},\;j=1,\;2,\;\ldots ,\;N^{+}$, and negative ones as $D_{i,k}^{+},\;k=1,\;2,\;\ldots ,\;N^{-}$, respectively. Please note that $N_{b}=N^{+}+N^{-}+1$. The batch similarity-based triplet loss for $x_{i}$ is defined as

(3) $$L_{trip}^{b}\left(D,x_{i}\right)=\max\left(0,m-\frac{1}{N^{+}}\sum_{j=1}^{N^{+}}{D_{i,j}^{+}}^{2}-\frac{1}{N^{-}}\sum_{k=1}^{N^{-}}{D_{i,j}^{-}}^{2}\right)$$

where m∈[0,1] is a constant. The two complex items in the brackets represent the average similarity for positive pairs and that for negative ones, respectively. Since $\left|D_{i,j}\right|\leq 1$, the square of $D_{i,j}$ is adopted instead of linear functions so that the loss can have a smoother gradient around the optimal solution. The average loss for the input batch is equal to the average of losses for every $x_{i}$, which is realized by

(4) $${\bar{L}}_{trip}^{b}=\frac{1}{N_{b}}\sum_{i=1}^{N_{b}}L_{trip}^{b}\left(D,x_{i}\right)$$

**Figure 3:**
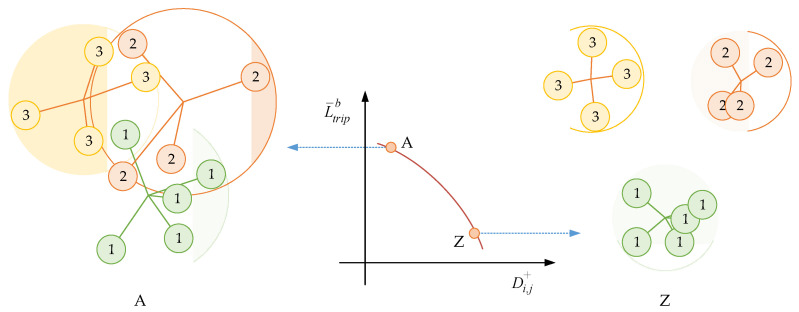
Diagram of clustering effect for batch similarity-based triplet loss.

As shown in Figure 3, by reducing the loss ${\bar{L}}_{trip}^{b}$, all the embedding vectors $f_{i}$ will move towards the center of the vectors $f_{i,j}^{+}$ and further away from the other kinds of vectors $f_{i,k}^{-}$. Because $D_{i,j}^{+}>0$ and $D_{i,k}^{-}<0$ are established, it can be inferred that the upper bound for ${\bar{L}}_{trip}^{b}$ is |1+m|. In addition, for a well-trained model, the lower bound for $D_{i,j}^{+}$ is m, while the allowed upper bound of $\left|D_{i,k}^{-}\right|$ is 1−m. In other words, m controls the strictness of the constraint for clustering, and it should be near 1. The reason for not setting m=1 directly is that it is unnecessary and unrealistic to let all the embedding vectors in the same class be the same. 

To accomplish the classification and evaluate the classification capability, the softmax classifier and the CE loss $L_{ce}$ are used. They are defined as

(5) $$p_{i,j}=\mathrm{softmax}\left(o_{i}\right)=\frac{e^{o_{i,j}}}{\sum_{j=1}^{C}e^{o_{i,j}}}$$

(6) $$L_{ce}=-\frac{1}{N_{b}}\sum_{i=1}^{N_{b}}\sum_{j=1}^{C}1_{y_{i}=j}\log p_{i,j}$$

where $o_{i,j}\in o_{i}$ is the output of the last fully connected layer in the model $F(x_{i})$, and $p_{i,j}$ is the predicted probability for sample $x_{i}$, belonging to the j-th class. $1_{y_{i}=j}$ represents that the ground truth $y_{i}$ is equal to j. The total loss is determined by both the CE loss and the BSTriplet loss as

(7) $$L=L_{ce}+\lambda {\bar{L}}_{trip}^{b}$$

where λ is a trade-off parameter. Since the value of ${\bar{L}}_{trip}^{b}$ is close to that of $L_{ce}$, the parameter λ is empirically set as 1.

### 2.3. Data Mining Strategy

For the regular triplet loss, each triplet must meet the requirement that every triplet input contains a sample $x_{i}$, and two reference samples $x_{i}^{+}$ and $x_{i}^{-}$ to form both a positive pair and a negative pair. Therefore, the data mining is necessary for constructing the available triplet input. In contrast with the regular triplet loss, the proposed BSTriplet loss will work well even in the case that samples in the batch can only compose positive pairs or negative ones. The only unavailable case for the BSTriplet loss is $N_{b}=1$. Nevertheless, to obtain the objective data distribution of the training dataset, we have designed a new data mining strategy, which can be described in the following steps:

1. Classify the original images into different categories according to the ground truth;
2. Cluster the original images into several groups for each category;
3. Count the number of images in each group, and calculate the ratio for each group;
4. Randomly select samples from every group to construct an input batch according to the obtained ratios.

The number of groups for each class in the dataset can be different because it depends on specific applications and data distribution. Notice that this strategy is only used in the training process. During the test phase of a trained CNN model, the model will ignore both the BSTriplet loss and the CE loss, and takes the output of the softmax function as the predicted results.

### 2.4. Computational Complexity

Supposing the number of training datasets is $N_{trn}$, it takes $N_{trn}$ floating point operations (FLOPs) for indexing in the first step of the proposed data mining scheme. As for the second step, its computational efficiency depends on the adopted clustering method. The required number of FLOPs for clustering is denoted as O(clsutering). Given the number g of groups of training images, the third step will take g FLOPs to obtain the ratios, and the fourth step needs $N_{b}$ FLOPs to construct an input batch. Notice that the first three steps of the proposed data mining strategy are carried out only once before the training process, and they need $O\left(clsutering\right)+N_{trn}+g$ FLOPs in total, while the fourth step will be performed once for each iteration in the training process.

The calculation process of the BSTriplet loss consists of basic mathematical operations. Therefore, it is computationally efficient and easy for implementation. Normalization for the embedding vectors requires $lN_{b}$ multiplication and $(l-1)N_{b}$ addition, which are $(2l-1)N_{b}$ FLOPs. Meanwhile, the calculation for obtaining the similarity matrix S needs $(l^{2}+l-1)N_{b}^{2}$ FLOPs. Besides this, encoding the ground truth y into a one-hot vector $\hat{y}$ takes $N_{b}$ operations of indexing and assignment, the calculation cost of which is considered to be $2N_{b}$ FLOPs in this paper for facilitating statistical analysis. The binary matrix B is generated with $(C^{2}+C-1)N_{b}^{2}$ FLOPs. As for the discriminative similarity matrix D, it is calculated once and shared for every sample in the batch, and it can be produced by setting the diagonal value of S⊗(2B−1) as 0. Therefore, the cost of D is $3N_{b}^{2}+N_{b}$ FLOPs. Equation (3) needs to be calculated repeatedly $N_{b}$ times, and it costs $2N_{b}^{2}+2N_{b}$ FLOPs in total. The average operation of the BSTriplet loss can be performed at a cost of $N_{b}$ FLOPs. Overall, the number of required FLOPs for the BSTriplet loss in each iteration is

(8) $$\mathrm{Cos}t\left({\bar{L}}_{trip}^{b}\right)=\left(l^{2}+C^{2}+l+C+3\right)N_{b}^{2}+(2l+5)N_{b}$$

## 3. Experimental Results

### 3.1. Experimental Setup

In the following, some experiments have been performed to test the effectiveness of the proposed BSTriplet loss. We have tried to apply the proposed loss to such popular light-weighted networks as EfficientNet-B1, MobileNet-V3-Small, ShuffleNet-V2 and PeleeNet for testing its performance. Those chosen models have distinctive structures and state-of-the-art performance, and they have been widely used in a variety of image classification tasks. The reason for adopting EfficientNet-B1 rather than the other models in [17] is that EfficientNet-B1 has a similar complexity to other light-weighted models, and its classification ability is better than that of the EfficientNet-B0. The number of parameters and the computational complexity of the compared networks are shown in Table 1, where the FLOPs are produced when the size of the input images is 128×128×1. According to Table 1, PeleeNet has the fewest parameters and MobileNet-V3-Small has the smallest number of FLOPs. All the involved networks are realized using Python 3.6.2 (downloaded from www.python.org) with Keras 2.3.1 and TensorFlow 2.0.0. In addition, all the following experiments are conducted on a computer with Ubuntu 16.04, a CPU of Intel Xeon Gold 6129 and a GPU of Nvidia Tesla V100 with CUDA 10.0 for acceleration. 

During the training process of every network, the initial learning rate is set as 0.001 to let the training loss get smaller quickly. The training loss is monitored. Once there is no improvement for the training loss for 20 epochs, the learning rate is multiplied by 0.3 to help the models find the optimal solutions. The maximum number of epochs is set as 400, and the minimum learning rate is set as $10^{-8}$. Besides this, some images are chosen from the test dataset to constitute the validation dataset for observing the performance of the network. In addition, the early-stopping technique is adopted to avoid the over-fitting problem, which is realized by stopping the training process once the improvement in validation loss is less than $10^{-4}$ for a successive 30 epochs.

### 3.2. The Influence of $N_{b}$

**Figure 4:**
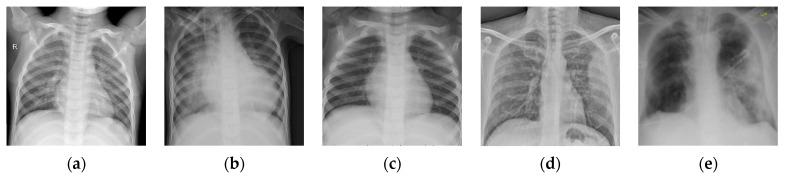
Examples of the Chest-1 dataset. (**a**) Normal; (**b**,**c**) pneumonia; (**d**,**e**) COVID-19.

The number of images in one batch $N_{b}$ is an important parameter in the calculation of the BSTriplet loss, because it can influence the similarity matrix S and further affect the estimation for training data distribution. Besides this, it has an impact on the stability of the training process. To explore the influence of $N_{b}$, we have downloaded a dataset of chest X-ray images [41,42] from the Kaggle [43] website. This dataset is denoted as “Chest-1” for brevity in the following. For this dataset, the X-ray images will be classified into three classes, including normal, (regular) pneumonia, and COVID-19. Several examples are shown in Figure 4. Here, these images are cropped to squares for better clarity. All the images are resized as 128×128 to be input into the network, and the construction of the Chest-1 dataset is shown in Table 2. According to the data mining strategy described above, we cluster the images in the Chest-1 dataset into six groups by K-means [44], wherein there are two groups for each class. Based on the ratios of groups, we have sampled each group to build different sizes of input batches $N_{b}\in \{6,12,18,24,30,36\}$. ModbileNet-V3-Small is used as the base framework and is trained by CE loss combined with the proposed BSTriplet loss. Some experiments have been performed with various $N_{b}$, while other hyper-parameters are fixed according to the method of controlling variables. The accuracy (ACC) is adopted as a metric to evaluate the performance, which is defined as:

(9) $$\mathrm{ACC}=\frac{\sum_{i=1}^{C}TP_{i}+\sum_{i=1}^{C}TN_{i}}{N^{\prime }}$$

where $TP_{i}$ and $TN_{i}$ are the numbers of true positive and true negative cases for the i-th class, respectively, and $N^{\prime }$ is the number of test images. 

**Figure 5:**
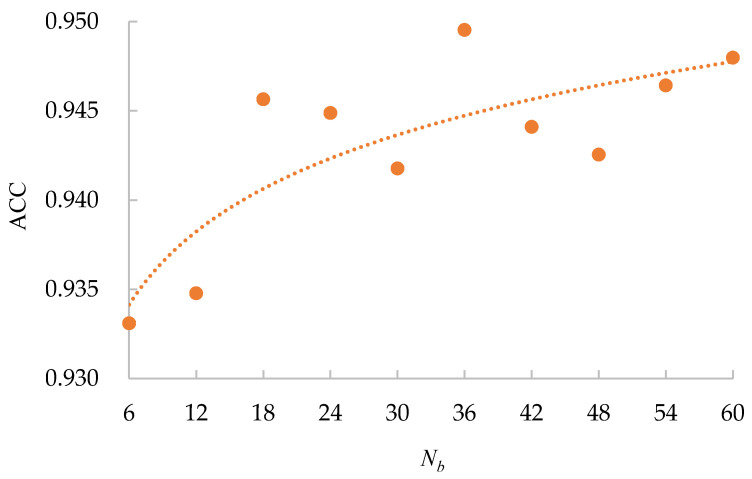
Accuracy of MobileNet-V3-Small for different $N_{b}$ on the Chest-1 dataset.

All the obtained ACC values for different $N_{b}$s are shown in Figure 5. From Figure 5, we can see that the accuracy (94.95%) achieved using $N_{b}=36$ is the highest, and the accuracy (93.31%) achieved using $N_{b}=6$ is the lowest. Overall, the accuracy has a positive correlation with $N_{b}$, which is in line with the supposition that a bigger $N_{b}$ will ensure that each input batch can represent the data distribution of the training dataset more precisely. Moreover, the curve converges quickly. The reason for this is that the CE loss provides a base accuracy, and it increases the stability of the curve. Furthermore, our data mining technique ensures the diversity of images in the batch. Therefore, a batch with a small $N_{b}$ has a similar data distribution to that with a big $N_{b}$. Considering that a bigger $N_{b}$ will lead to insufficient iteration times for network training in one epoch, we will use $N_{b}=36$ as the default setting for the rest of the experiments.

### 3.3. Clustering Effect of the BSTriplet Loss

**Figure 6:**
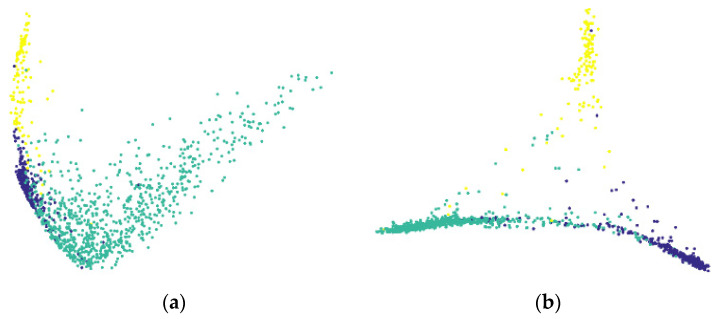
The visualization of the embedding vectors obtained by MobileNet-V3-Small trained with different kinds of loss functions. (**a**) Cross entropy; (**b**) cross entropy combined with BSTriplet.

To display the effect of the BSTriplet loss in an intuitive way, principal component analysis (PCA) was applied to reduce the dimension of the embedding vectors for visualization. We have compared two distributions of embedding vectors for the training images in Figure 6, which are generated via two kinds of MobileNet-V3-Small trained with different kinds of loss function for 10 epochs using the batch size $N_{b}=36$. For all the obtained embedding vectors, their dimensions are reduced from 1280 to 2 through PCA. Figure 6a is obtained via the model trained by only CE loss, while Figure 6b involves the BSTriplet loss. Obviously, the BSTriplet loss is able to increase the inter-class distance and decrease the intra-class distance. In other words, it helps to improve the classification ability of MobileNet-V3-Small.

### 3.4. Effect of the Data Mining Strategy

**Figure 7:**
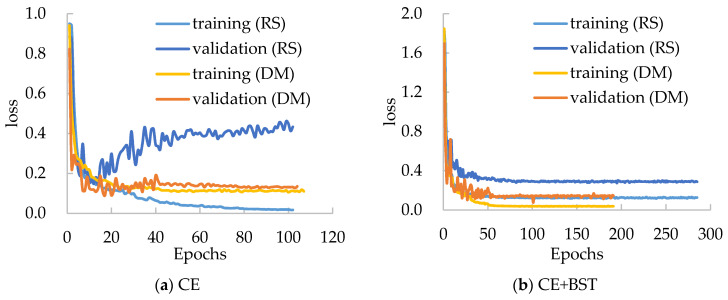
Loss curves of different training schemes for MobileNet-V3-Small on the Chest-1 dataset.

To assess the effect of the data mining strategy, we trained MobileNet-V3-Small with several schemes. The training schemes utilize random selection (RS) or the proposed data mining (DM) strategy to construct input batches, and use CE or “CE+BST” as the loss function, where the latter means CE combined with the BSTriplet function. The loss curves of training and validation for each training scheme are given in Figure 7, and the obtained ACC values are shown in Table 3. In Figure 7, “RS” and “DM” refer to the schemes using the random selection and the proposed data mining strategy, respectively. From Figure 7a, we can see that the loss curves of the DM scheme are much smoother than those of the RS scheme. This indicates that the network is easier to train using the date mining strategy. Besides this, for two kinds of loss functions, the proposed strategy can reduce the gap between the training loss and the validation loss, which means that it can alleviate the over-fitting problem.

From Table 3, it can be seen that the data mining strategy used in the CE-based model can improve the accuracy by 1.21% compared with random selection. For the model based on CE+BST loss, its accuracy is improved by 0.90%. The results show that the proposed data mining strategy is helpful for training CNN models. Here, the strategy is more helpful for the CE-based model than for the CE+BST based one. The reason is that the BSTriplet loss can evaluate the data distribution of the training dataset to a certain extent, while the CE function lacks this ability. 

### 3.5. Applicability of the BSTriplet Loss

**Figure 8:**
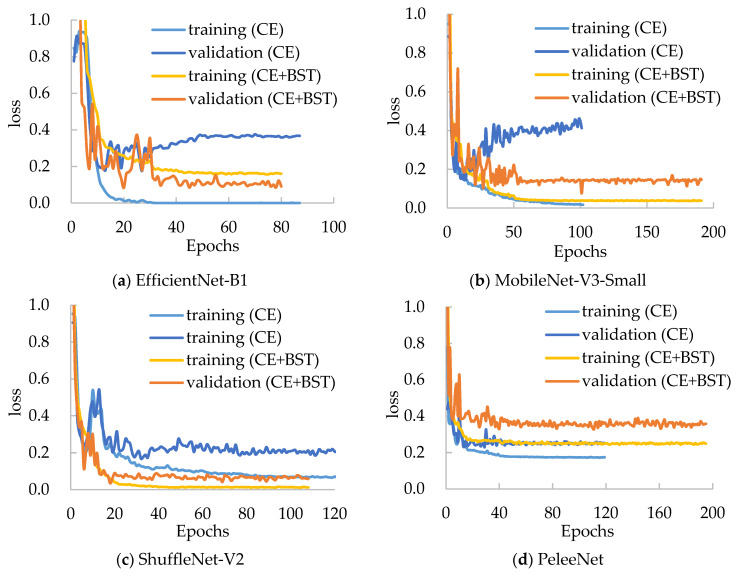
Loss curves for the compared networks on the Chest-1 dataset.

Moreover, we have compared the performances of several mentioned models with the BSTriplet loss to discover its applicability and effect. For each compared network, we have trained it with the CE loss and CE+BST loss. The training loss and validation loss are shown in Figure 8. By comparing the four networks trained with CE loss, we can see that the validation loss of EfficientNet-B1 and MobileNet-V3-Small goes up when the time of iteration gets bigger. This observation means that there is a more severe over-fitting problem in their training process compared with the others. When these models are trained by CE+BST loss, the over-fitting problem is suppressed. Moreover, the gap between training loss and validation loss demonstrates that there is a smaller gap for CE+BST loss than for CE loss in such models as EfficientNet-B1, MobileNet-V3-Small and ShuffleNet-V2. As for PeleeNet, it has a relatively small over-fitting problem, which benefits from the fact that it has the lowest number of weights. Overall, the BSTriplet loss can suppress the over-fitting problem, which demonstrates that the BSTriplet loss can play the role of a regularization term for the CE loss.

To verify the effectiveness of the proposed combined loss, we have compared it with CE combined with triple loss [45] and CE combined with the improved lifted structure loss [46]. We test all these loss functions on four compared networks on the Chest-1 dataset. Since Chest-1 provides a multi-class classification task, the average sensitivity $\bar{\mathrm{SEN}}$ and specificity $\bar{\mathrm{SPE}}$ are used as metrics for evaluating the performance of the networks. $\bar{\mathrm{SEN}}$ and $\bar{\mathrm{SPE}}$ are calculated as

(10) $$\bar{\mathrm{SEN}}=\frac{1}{C}\sum_{i=1}^{C}{\mathrm{SEN}}_{i},{\;\mathrm{with}\;\mathrm{SEN}}_{i}=\frac{TP_{i}}{TP_{i}+FN_{i}}\in [0,1]$$

(11) $$\bar{\mathrm{SPE}}=\frac{1}{C}\sum_{i=1}^{C}{\mathrm{SPE}}_{i},{\;\mathrm{with}\;\mathrm{SPE}}_{i}=\frac{TN_{i}}{TN_{i}+FP_{i}}\in [0,1]$$

where ${\mathrm{SEN}}_{i}$ and ${\mathrm{SPE}}_{i}$ respectively represent the sensitivity and specificity of the i-th class, which shows the ability of the classifier to correctly find real positive cases and negative ones for the target disease; $FP_{i}$ and $FN_{i}$ denote the number of false positive and false negative cases for the i-th class, respectively. Besides this, the ACC and the area under the curve (AUC) of the receiver operating characteristic (ROC) are also employed to assess the classification ability of the trained models. The results are listed in Table 4, where “Triplet”, “LS” and “BST” represent the regular triplet loss, the improved lifted structure loss and the proposed BSTriplet loss, respectively. For each evaluated model with different losses, the best value for every metric is indicated with bold in Table 4. Clearly, ShuffleNet-V2 provides the highest accuracy among all the compared networks for each of the four different losses, which demonstrates the superiority of its structure. For each network alone, both the LS loss and the BSTriplet loss can improve the accuracy compared with the CE loss. By comparison, the regular triplet loss causes ACC to decrease from 92.70% to 92.24% for MobileNet-V3-Small, and ACC to decrease from 92.93% to 90.06% for PeleeNet. The reason is that the regular triplet loss identifies each input sample only according to one positive pair and one negative pair, which easily leads to an unstable training process and could make it difficult to search for the optimal solution. Furthermore, among all the compared losses, the CE+BST loss guides such models as EfficientNet-B1, MobileNet-V3-Small and ShuffleNet-V2 to gain the highest accuracy, sensitivity, specificity and AUC values. As for PeleeNet, the proposed CE+BST loss achieves the second highest AUC value (0.9841) and specificity (96.21%). Overall, the BSTriplet loss is helpful for training light-weighted CNN models when employed and combined with CE loss, and it is more effective than the regular triplet loss and lifted structure loss.

**Figure 9:**
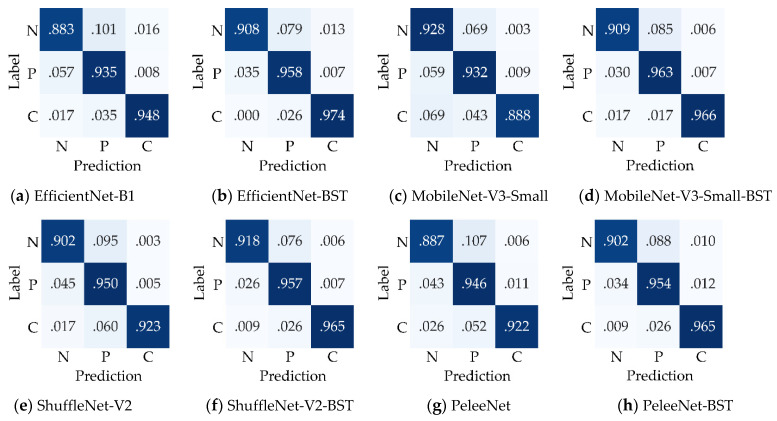
Confusion matrixes of the compared networks trained with CE loss or CE+BST loss on the Chest-1 dataset. “N”, “P” and “C” denote normal images, pneumonia images and COVID-19 images, respectively.

To intuitively show the superiority of the proposed BSTriplet loss, the confusion matrixes of the four networks trained with CE loss and CE+BST loss are given in Figure 9. It can be seen that the normal images are relatively more easily misclassified as pneumonia images or COVID-19 images for all the compared models. Therefore, the proposed loss has the lowest average recognition rate (0.905) over all trained models. However, the average recognition rates for pneumonia images and COVID-19 images are 0.949 and 0.944, respectively. In addition, EfficientNet-B1, ShuffleNet-V2 and PeleeNet achieve higher recognition rates for every class when trained with CE+BST loss compared to when trained with CE loss. As for the MobileNet-V3-Small model, the proposed CE+BST loss causes the recognition rate for normal images to decrease from 0.928 to 0.909, but it has a much better ability to distinguish the images of the rest of the categories. Moreover, the recognition rate for COVID images is improved the most among the three classes when the BSTriplet loss is used, which indicates that it can resolve the problem of data imbalance.

**Figure 10:**
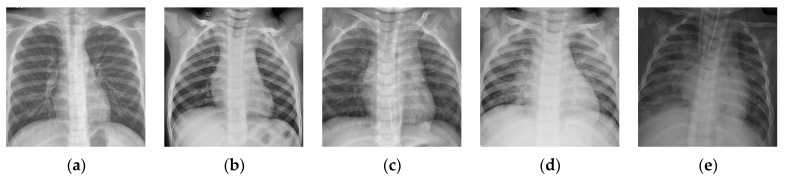
Examples of the dataset Chest-2. (**a**–**c**) Normal; (**d**,**e**) pneumonia.

To further verify the consistency of the proposed BSTriplet loss, we have carried out comparative experiments on another dataset of chest X-ray images denoted as “Chest-2”. This dataset is also downloaded from the Kaggle [43] website. Here, Chest-2 is used for the classification of lung images into normal lung images and pneumonia images. Some examples are shown in Figure 10. The construction of Chest-2 is listed in Table 5. Each of the mentioned four light-weighted networks is trained with four kinds of losses on Chest-2. Similar to the experiments on Chest-1, all these images in Chest-2 are resized into 128×128. We have performed the K-means algorithm to partition the training images into six groups, and randomly selected six samples from each group to build the input batch for the proposed CE+BST loss. 

**Figure 11:**
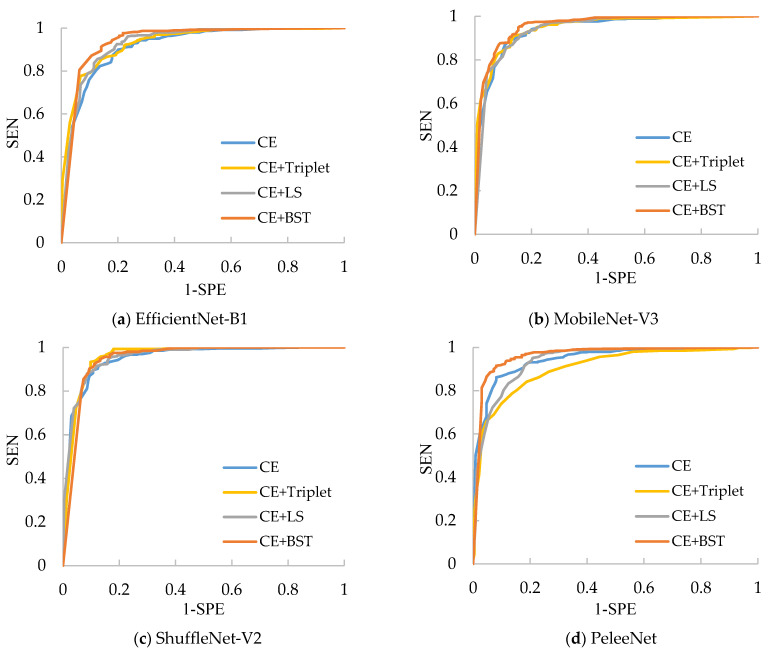
ROC curves for the compared networks with different losses on the Chest-2 dataset.

The results for the classification of Chest-2 are shown in Table 6. From Table 6, we can see that ShuffleNet-V2 still has the best performance because it can achieve the highest average SEN (97.14%), average SPE (80.56%), average ACC (90.91%) and average AUC (0.9569) over four kinds of losses. Besides this, the CE+Triplet loss helps ShuffleNet-V2 to provide the highest ACC (92.47%), which is 3.21% higher than that of ShuffleNet-V2 trained with CE loss. Nevertheless, CE+Triplet loss achieves lower ACC and AUC compared with CE loss when they are applied to PeleeNet, which reveals that the consistency of the effectiveness of CE+Triplet loss cannot be guaranteed for various networks. In comparison, both CE+LS and our CE+BST loss have better consistency, which benefits from the analysis for all the pairs formed by the samples in the batch. Furthermore, the proposed CE+BST loss surpasses the CE+LS loss in terms of average values of SEN, SPE, ACC and AUC over four evaluated networks by 0.26%, 7.27%, 2.89% and 0.012%, respectively. The reason why BSTriplet outperforms LS is that the former adopts similarity instead of Euclidean distance, so that it has a clear upper bound, and the value of BSTriplet loss is close to CE loss, so that it can achieve better coordination than LS loss. We have also provided the ROC curves of all compared methods in Figure 11. When the ROC curve is closer to the upper left corner, it means the corresponding classifier has a better classification ability. For ShuffleNet-V2, all the three combined losses show similar performances, and they all surpass the performance of CE loss. As for the rest of the networks, our CE+BST loss can provide the most significant improvement in classification performance for each network, especially when it is used in PeleeNet. In general, the proposed BSTriplet loss is more suitable for assisting CE loss in CNN training, and it outperforms the other compared DML losses.

**Figure 12:**
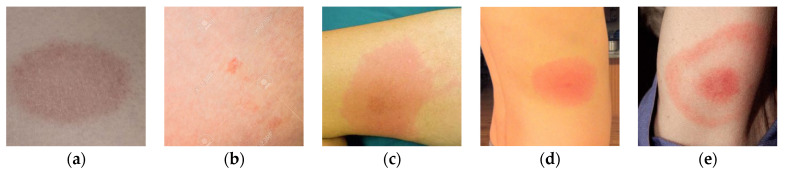
Examples of rash images. (**a**,**b**) Non-Lyme disease; (**c**–**e**) Lyme disease.

To further validate the effectiveness of the proposed BSTriplet loss, some experiments have been conducted on a skin rash image dataset, which is used to distinguish the images containing Lyme disease from those with other disease [47]. The composition of the rash image dataset is listed in Table 7. Figure 12 shows some images in the rash image dataset. It can be seen that the images in this dataset are colorful optical images, which are much different from the above X-ray images. Besides this, the number of images in the rash image dataset is evidently less than that in the above datasets. To alleviate the over-fitting problem, we have augmented the training images by such methods as rotation by $90^{{}^{\circ}}$, $180^{{}^{\circ}}$ and $270^{{}^{\circ}}$, horizontal/vertical flipping, and horizontal/vertical translation for five pixels so that the number of training images is enlarged by seven-fold. As for the test images, the augmentation has not been implemented. We have clustered the images into four categories, and built the input batches for the proposed CE+BST loss according to the steps in Section 2.3. 

The results for each evaluated model are given in Table 8. It can be seen that almost all the metrics are lower than those in the above experiments. The reason for this is that the rash images are full of varied backgrounds and the training images are insufficient. Nevertheless, when the loss involves the DML methods, the CNNs can gain higher accuracy compared with themselves trained with CE loss, except that CE+LS loss provides the same ACC (75.86%) for MobileNet-V3-Small. MobileNet-V3-Small provides the highest ACC (83.91%) and AUC (0.8755) when trained with the proposed CE+BST loss, and it can produce the second best ACC (82.76%) and AUC (0.8423) when it is trained with CE+Triplet loss. Both ShuffleNet-V2 and PeleeNet gain the second highest ACC (80.46%) when they are trained with our CE+BST loss. A comparison among the four results obtained by PeleeNet shows that CE+LS loss gives the highest SEN but the lowest SPE, while CE+Triplet loss gives the opposite results. In comparison, our CE+BST loss can provide both the second highest SEN (77.78%) and SPE (82.35%), as well as the highest ACC (80.46%), for PeleeNet. Besides this, if the results for the CE loss are used as the baseline for each model, the average improvement for each combined loss in four models can be calculated. The proposed CE+BST loss gains an advantage over CE+Triplet loss and CE+LS loss by providing the highest average improvements in SEN, SPE, ACC and AUC, by 9.03%, 5.39%, 6.90% and 0.1063, respectively.

**Figure 13:**
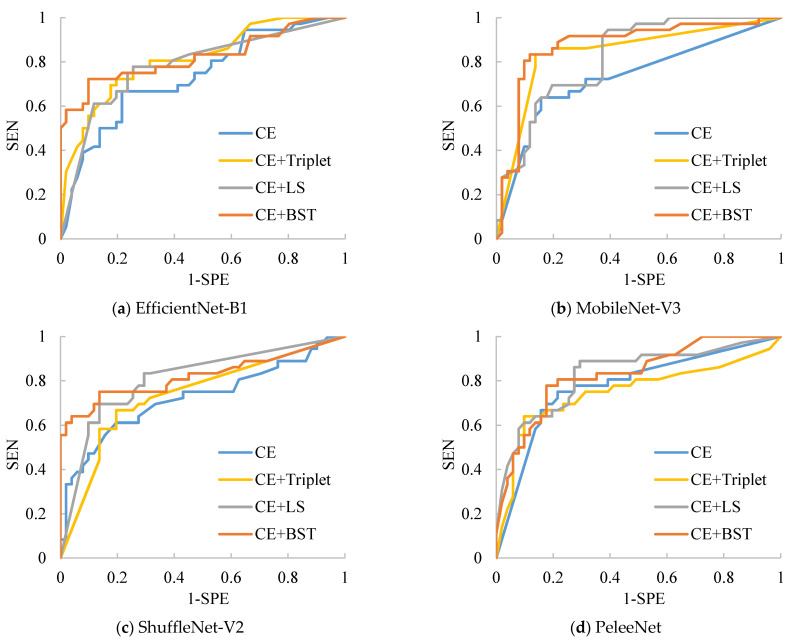
ROC curves for the compared networks with different losses on the rash image dataset.

The ROC curves for every test model on the rash image dataset are shown in Figure 13. It is clear that these curves are not as smooth as those in Figure 11 due to the small number of test images. Almost all the combined losses surpass CE loss for each compared CNN, especially for MobileNet-V3-Small. Only CE+Triplet is inferior to CE loss when applied to PeleeNet, which reveals its instability again. In addition, the curves of the proposed CE+BST loss are much closer to the top left corner in each subfigure than the rest, which demonstrates its effectiveness for a small dataset and adaptability to different networks. 

**Figure 14:**
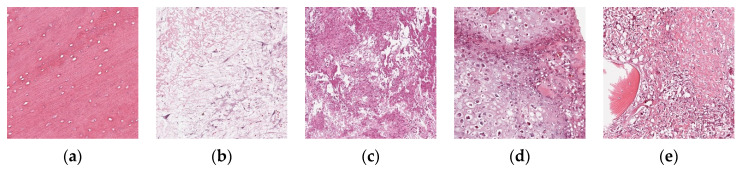
Examples of the osteosarcoma histology images. (**a**) Non-tumor; (**b**,**c**) necrotic tumor; (**d**,**e**) viable tumor.

**Figure 15:**
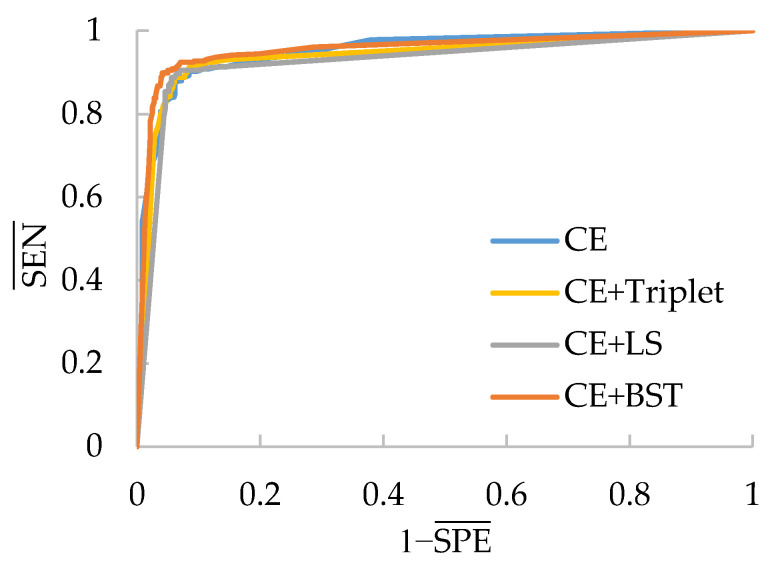
ROC curves of MobileNet-V3-Small with different losses on the osteosarcoma histology image dataset.

To further verify the applicability of the BSTriplet loss to other medical image modalities, additional experiments have been done on an osteosarcoma histology image dataset [48,49,50], which can be accessed from the cancer imaging archive (TCIA) [51]. There are three kinds of images in the osteosarcoma histology image dataset, as shown in Figure 14. Compared with the above involved images, the histology images are colored images, and their backgrounds are simpler than those of the skin rash images. This dataset is a small sample dataset and its construction is listed in Table 9. The augmentation for this dataset is the same as that for the rash images. We have clustered each kind of images into two groups for CE+BST loss according to the proposed data mining strategy. Using the accuracy ACC, average sensitivity $\bar{\mathrm{SEN}}$, average specificity $\bar{\mathrm{SPE}}$ and AUC as metrics, we have tested MobileNet-V3-Small trained with several different loss functions. The results are provided in Table 10 and Figure 15. 

Table 10 shows that CE+BST loss-based MobileNet-V3-Small provides the best performance in terms of all the metrics. In particular, the accuracy provided by CE+BST loss is 2.18% higher than the second best ACC, which is produced by CE+LS loss. Besides this, the regular triplet loss and the LS loss help the CE to obtain better $\bar{\mathrm{SEN}}$, $\bar{\mathrm{SPE}}$ and ACC values, although their AUC values are smaller than those of CE loss. On the other hand, BSTriplet loss obtains the best AUC value in Table 10 and ROC curve in Figure 15. The results demonstrate that, compared with the LS loss and the regular triplet loss, our proposed BSTriplet loss has a better ability to assist CE loss in improving the performance of the CNNs on small sample datasets.

## 4. Conclusions

In this paper, a novel batch similarity-based triple loss is proposed for light-weighted CNNs in the case of medical image classification. The proposed loss takes the similarities among all the samples in the input batch into account in order to gather samples of the same class and distinguish those of different classes. It can be easily assembled into existing CNNs, and assist cross entropy loss in training the CNNs by resolving the over-fitting problem. A reasonable data mining technique is also provided, which can help to build input batches according to the distribution of the training data. Several experiments have been implemented on such medical image datasets as chest X-ray images and rash images. The results show the superiority and consistency of the proposed loss combined with cross entropy loss compared to other combined losses in terms of sensitivity, specificity, accuracy and AUC. Our further work will be focused on the optimization of the computational efficiency of the proposed loss, its combination with more loss, and testing on other image datasets to ensure that the training process of CNNs will be more stable and the over-fitting problem can be addressed more effectively. 

## Figures and Tables

**Table 1:** Complexity of the compared networks.

Metrics	EfficientNet-B1	MobileNet-V3-Small	ShuffleNet-V2	PeleeNet
Parameters (M)	6.58	3.04	4.02	2.11
FLOPs (M)	395	43.7	319	323

**Table 2:** The construction of the Chest-1 dataset.

	Normal	Pneumonia	Covid-19	Total
Training	1266	3418	920	5604
Testing	317	855	116	1288
Total	1583	4273	1036	6892

**Table 3:** Accuracy of different training schemes for MobileNet-V3-Small on the Chest-1 dataset.

Schemes	CE (RS)	CE (DM)	CE+BST (RS)	CE+BST (DM)
ACC (%)	92.70	93.91	94.05	94.95

**Table 4:** Metrics for the compared networks on the Chest-1 dataset.

Model	Loss	$\bar{\mathrm{SEN}}\;(\%)$	$\bar{\mathrm{SPE}}\;(\%)$	ACC (%)	AUC
EfficientNet-B1	CE	92.20	95.14	92.31	0.9523
	CE+Triplet	94.28	96.10	94.18	0.9613
	CE+LS	91.67	94.86	93.32	0.9406
	CE+BST	**94.69**	**96.53**	**94.72**	**0.9849**
MobileNet-V3-Small	CE	91.58	95.67	92.70	0.9795
	CE+Triplet	91.80	95.04	92.24	0.9741
	CE+LS	92.71	95.66	93.40	0.9792
	CE+BST	**94.55**	**96.58**	**94.95**	**0.9807**
ShuffleNet-V2	CE	92.48	95.60	93.56	0.9780
	CE+Triplet	94.21	96.63	94.95	0.9800
	CE+LS	94.75	96.75	95.19	0.9791
	CE+BST	**95.02**	**96.90**	**95.50**	**0.9861**
PeleeNet	CE	91.83	95.23	92.93	0.9788
	CE+Triplet	90.03	94.21	90.06	0.9673
	CE+LS	90.85	**96.50**	93.48	**0.9882**
	CE+BST	**94.07**	96.21	**94.25**	0.9841

**Table 5:** The construction of the Chest-2 dataset.

Data	Normal	Pneumonia	Total
Training	1341	3875	5216
Testing	234	390	624
Total	1575	4265	5840

**Table 6:** Metrics for the compared networks on the Chest-2 dataset.

Model	Loss	SEN (%)	SPE (%)	ACC (%)	AUC
EfficientNet-B1	CE	93.85	72.65	85.90	0.9178
	CE+Triplet	94.87	72.22	86.38	0.9323
	CE+LS	96.67	72.65	87.66	0.9327
	CE+BST	**97.69**	**77.35**	**90.06**	**0.9457**
MobileNet-V3-Small	CE	96.92	70.51	87.02	0.9424
	CE+Triplet	93.08	81.20	88.62	0.9486
	CE+LS	**97.18**	71.37	87.50	0.9441
	CE+BST	94.87	**84.62**	**91.03**	**0.9603**
ShuffleNet-V2	CE	95.64	78.63	89.26	0.9535
	CE+Triplet	**99.23**	81.20	**92.47**	**0.9593**
	CE+LS	96.15	80.77	90.38	0.9569
	CE+BST	97.44	**81.62**	91.51	0.9579
PeleeNet	CE	95.13	70.51	85.90	0.9472
	CE+Triplet	88.72	73.50	83.01	0.9057
	CE+LS	94.36	73.50	86.54	0.9458
	CE+BST	**95.38**	**83.76**	**91.03**	**0.9649**

**Table 7:** The construction of the rash image dataset.

Data	Non-Lyme Disease	Lyme Disease	Total
Training	206	151	357
Testing	51	36	87
Total	257	187	444

**Table 8:** Metrics for the compared networks on the rash image dataset.

Model	Loss	SEN (%)	SPE (%)	ACC (%)	AUC
EfficientNet-B1	CE	66.67	78.43	73.56	0.7334
	CE+Triplet	72.22	78.43	75.86	0.8132
	CE+LS	72.22	76.47	74.71	0.7835
	CE+BST	**75.00**	**82.35**	**79.31**	**0.8227**
MobileNet-V3-Small	CE	63.89	84.31	75.86	0.7323
	CE+Triplet	**83.33**	82.35	82.76	0.8423
	CE+LS	61.11	86.27	75.86	0.8224
	CE+BST	72.22	**92.16**	**83.91**	**0.8755**
ShuffleNet-V2	CE	61.11	80.39	72.41	0.7230
	CE+Triplet	66.67	80.39	74.71	0.7358
	CE+LS	69.44	**86.27**	79.31	0.8088
	CE+BST	**75.00**	84.31	**80.46**	**0.8208**
PeleeNet	CE	77.78	72.55	74.71	0.7764
	CE+Triplet	63.89	**90.20**	79.31	0.7514
	CE+LS	**83.33**	72.55	77.01	**0.8292**
	CE+BST	77.78	82.35	**80.46**	0.8287

**Table 9:** The construction of the osteosarcoma histology image dataset.

	Non-Tumor	Necrotic Tumor	Viable Tumor	Total
Training	429	210	276	915
Testing	107	53	69	229
Total	536	263	345	1144

**Table 10:** The metrics for MobileNet-V3-Small on the osteosarcoma histology image dataset.

Model	Loss	$\bar{\mathrm{SEN}}\;(\%)$	$\bar{\mathrm{SPE}}\;(\%)$	ACC (%)	AUC
MobileNet-V3-Small	CE	87.66	93.80	87.34	0.9508
	CE+Triplet	88.17	94.15	87.77	0.9391
	CE+LS	88.72	94.35	88.21	0.9265
	CE+BST	**90.48**	**95.45**	**90.39**	**0.9549**

## Data Availability

Publicly available datasets were analyzed in this study. The Chest-1 dataset presented in this study can be found here: https://www.kaggle.com/prashant268/chest-xray-covid19-pneumonia. The Chest-2 dataset is openly available in the Mendeley Data at http://dx.doi.org/10.17632/rscbjbr9sj.2, and it can also be accessed in the Kaggle website at https://www.kaggle.com/paultimothymooney/chest-xray-pneumonia. The skin rash image dataset is openly available in Kaggle at https://www.kaggle.com/sshikamaru/lyme-disease-rashes. The osteosarcoma histology image data can be found in The Cancer Imaging Archive (TCIA) at https://doi.org/10.7937/tcia.2019.bvhjhdas.
